# High-performance flexible metal-on-silicon thermocouple

**DOI:** 10.1038/s41598-018-32169-9

**Published:** 2018-09-13

**Authors:** Daniel Assumpcao, Shailabh Kumar, Vinayak Narasimhan, Jongho Lee, Hyuck Choo

**Affiliations:** 1 Department of Electrical Engineering, California Institute of Technology, Pasadena, CA 91125 United States; 2 Department of Medical Engineering, California Institute of Technology, Pasadena, CA 91125 United States; 3 School of Mechanical Engineering, Gwangju Institute of Science and Technology, Gwangju, Republic of Korea

## Abstract

We have demonstrated metal-on-silicon thermocouples with a noticeably high Seebeck coefficient and an excellent temperature-sensing resolution. Fabrication of the thermocouples involved only simple photolithography and metal-liftoff procedures on a silicon substrate. The experimentally measured Seebeck coefficient of our thermocouple was 9.17 × 10^−4^ V/°K, which is 30 times larger than those reported for standard metal thin-film thermocouples and comparable to the values of alloy-based thin-film thermocouples that require sophisticated and costly fabrication processes. The temperature-voltage measurements between 20 to 80 °C were highly linear with a linearity coefficient of 1, and the experimentally demonstrated temperature-sensing resolution was 0.01 °K which could be further improved up to a theoretical limit of 0.00055 °K. Finally, we applied this approach to demonstrate a flexible metal-on-silicon thermocouple with enhanced thermal sensitivity. The outstanding performance of our thermocouple combined with an extremely thin profile, bending flexibility, and simple, highly-compatible fabrication will proliferate its use in diverse applications such as micro-/nanoscale biometrics, energy management, and nanoscale thermography.

## Introduction

Accurate thermal measurements serve crucial roles in monitoring industrial processes as well as understanding various natural phenomena^1,2^. With recent improvements in the fabrication of small-scale devices, there is a renewed interest in technologies for studying thermal phenomena at micro- and nanoscales^3–6^. High-resolution detection of temperature changes on small scales could open up new scientific possibilities by providing valuable insight into the thermal energy distribution due to near-field enhancement in nanophotonic structures and mapping subtle temperature differences in biological samples to reveal functional details of cell biology or simply the health of the cells. For example, a locally elevated temperature observed in an atherosclerotic plaque could indicate inflammation that could lead to a potentially fatal rupture^7–11^. Achieving higher thermal sensing resolution in compact forms can also lead to cost-effective and compact temperature-monitoring modules in mobile applications such as artificial skin, smart bandages, and other wearable sensors^12^.

For micro- and nanoscale thermal mapping, various measurement techniques such as thermocouples^10,13,14^, infrared thermography^15^, optical interferometry^1^, and fluorescence and luminescence thermography^2,3,9,16^ have been utilized^3,4,9,16^. While optical approaches allow remote temperature sensing, their resolutions are fundamentally limited by the diffraction of light, and their use can be challenging in a light-scattering environment such as in biological tissue^2,4,17^. In addition, properly interpreting the measurement data produced by some optical methods such as infrared tomography or optical interferometry requires sufficient prior knowledge of the optical properties of the complex biomedical environments involved in the measurement^4^. Furthermore, fluorescence and luminescence thermography require *in-situ* placement of temperature-sensitive molecules, which can be inconvenient and invasive^4,10^.

Thermocouples, on the other hand, utilize the Seebeck effect and directly convert a local temperature to an electrical voltage. They have been widely used in diverse systems for more than a century due to their simple fabrication, ease of use, and accuracy^10,13,14^. A standard thermocouple is made up of two arms, consisting of conductors, semiconductors, or a combination of the two, which intersect at the measurement point. A voltage proportional to the temperature difference between the intersecting measurement point and the open ends is generated across the arms^18^ and measured from the open ends. The magnitude of this thermoelectric conversion is represented by a proportionality constant called the Seebeck coefficient (S).

Thermocouples can be easily fabricated using standard micro- and nano-fabrication and process-compatible thin metallic films such as Ni, Cr, or Au layers, and they have been demonstrated for various applications^5,6,14,19,20^. However, these process-compatible metals possess small Seebeck coefficients (Table 1)^18^, and their poor sensitivity remains a major drawback, yielding temperature-sensing resolutions typically larger than 2 °C (Table 1). Improving their thermal sensitivity requires the use of costly and bulky sophisticated measurement electronics or alloys with high Seebeck coefficients such as bismuth and antimony telluride that are rare, expensive, and challenging to fabricate using standard processes^21^. Semiconductors such as silicon (Si) are also known to have high Seebeck coefficients^19^, but previous studies have primarily focused on interaction between metal films and semiconductors with respect to their thermoelectric properties^22–24^. In addition, the inherent high resistance of silicon decreases the final thermal sensitivity and has prevented their utilization in practical thermocouple applications^25–27^. Furthermore, previously demonstrated silicon-based thermocouples involved challenging fabrication processes such as elaborate wafer-etching and bonding to accomplish electrical isolations between the silicon and metal as well as to demonstrate compatibility with bipolar or CMOS processes^28^.

**Table 1:** List of recently reported thin-film thermocouples and their corresponding Seebeck coefficients.

Thermocouple Materials	Seebeck Coefficient (µV/K)	Typical Sensitivity (°C)
Cr-Ni Array^46^	26.5	2.3
Ni Array^19^	1.06	57
Cr-Pt Array^20^	14.9	4.0
Bi_2_Te_3_ – Sb_2_Te_3_^21^	360	0.17
Al-pSi^27^	1170	0.05
Our Cr-Si thermocouple	924	0.07

Typical sensitivities were calculated assuming a minimum voltage-detection level of 60 µV.

**Figure 1:**
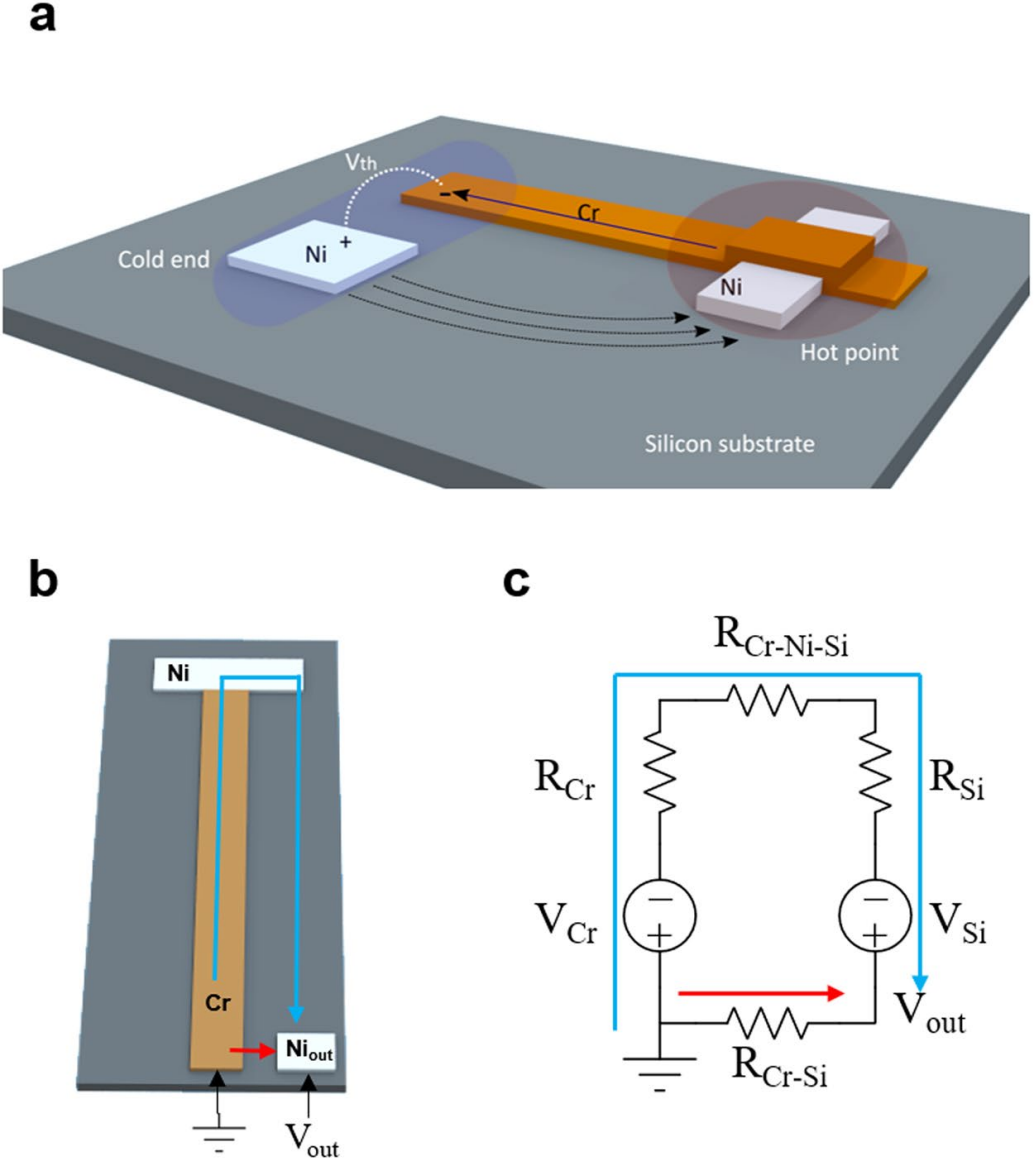
(**a**) Overview of the thermocouple structure. The thermally generated voltage (V_th_) is measured at the cold end. The chrome strip forms a Schottky barrier and stays electrically isolated from the silicon substrate except at the hot point where the rectangular nickel pad forms a conductive ohmic contact and connects the chrome arm electrically to the silicon substrate only at the hot point, forming the chrome-silicon junction, resulting in a chrome-on-silicon thermocouple that makes the excellent use of the silicon’s large Seebeck coefficient while keeping the fabrication process simple. (**b**) Overall schematic of the thermocouple with the involved current path drawn on it. The colored arrows indicate the current path. (**c**) Equivalent resistive circuit for the chrome-on-silicon thermocouple in (**b**) to study their effects on the final Seebeck coefficient. The colored arrows indicate the current paths and the voltage measured between the points is a weighted average of the voltage change on each path. The blue arrow corresponds to the ideal current path leading to the highest output voltage consisting of the sum of the two Seebeck voltages. The red arrow corresponds to the very lossy current path leading to no voltage output (R_Cr_: the resistance of the Cr arm; R_Si_: the resistance of the silicon between the measurement point and the pad; R_Cr-Si_: the isolation resistance of the Schottky barrier between the Cr arm and the silicon; R_Cr-Ni-Si_: the resistance of the Ni ohmic contact pad between the chromium and silicon arm; V_out_: the output voltage measured at the pads; V_Cr_: the Seebeck voltage contributed by the chromium arm; and V_Si_: the Seebeck voltage contributed by the silicon).

Using single-crystal-silicon wafers and two straightforward fabrication processes, we have demonstrated microscale metal-on-silicon thermocouples with sensitivities 30 times higher than previously reported metal-film-based thermocouples (Fig. 1(a)). This closely matches the best performance of the alloy-based thermocouples. In order to take full advantage of the high Seebeck coefficient of silicon, we minimized the internal resistive loss in silicon by utilizing the larger current-conducting cross-section of the silicon substrate. In addition, we kept the fabrication process simple by choosing appropriate thermocouple materials and utilizing Schottky barriers and ohmic contacts to create electrical isolation and contacts at appropriate places. This produced metal-on-silicon thermocouples with an experimentally measured sensitivity of 0.01 °K and a theoretical thermal sensitivity down to 5.5×10^-4^ K on a silicon substrate fabricated in two processing steps.

Development of flexible sensors is another rapidly growing field with relevance in diverse areas including thermography^29^, photodetection^30^, and wearable devices^12,31–34^ for biomedical applications. Thermal sensitivity is an important attribute for wearable technologies such as electronic artificial skin for monitoring body temperature as well as wound healing^12,33,35,36^. Wafer-thinning approaches have been applied in the past for fabrication of flexible electronics using silicon wafers^37,38^. We utilized this wafer-thinning fabrication design and combined it with our sensor design to demonstrate an array of highly flexible thermocouples with excellent temperature-sensing resolution.

## Results/Discussion

### Design and Modeling of Si-Metal Thermocouples with High Seebeck Coefficient

Our design utilizes a thin metal film as one of the thermocouple arms and a single-crystal silicon substrate as the other arm to take full advantage of the large Seebeck coefficient of silicon. To implement this approach without adding complex fabrication processes, we created an electrically isolated metal arm on a single crystal silicon substrate through the formation of a Schottky barrier. This also minimizes the inherent resistive loss in the silicon by utilizing a large cross-sectional area of the substrate for current conduction, resulting in an undiminished final Seebeck coefficient.

We chose chromium with a work function of 4.5 eV as the material for the metal arm and a properly doped p-type silicon substrate (conductivity: 1–10 Ω cm) with a work function of 5 eV to serve as a silicon arm, so that an electrically isolating Schottky barrier is formed between them. The two arms, chromium and silicon, were joined electrically only at the hot point through a thin-film nickel pad with a work function of 5.15 eV which formed an ohmic contact with both the chromium metal film and the p-type silicon substrate. (See S1 in supplementary information (SI) for more details).

To optimize the design of the proposed chrome-on-silicon thermocouple and maximize the final Seebeck coefficient, we performed circuit analysis as shown in Fig. 1(b,c). We obtained the following equation for the Seebeck coefficient of the thermocouple in terms of the material resistances:

1 $${{S}}_{{eff}}=\frac{{{R}}_{{Cr}-{Si}}}{{{R}}_{{Cr}-{Si}}+({{R}}_{{Cr}}+{{R}}_{{Cr}-{Ni}-{Si}}+{{R}}_{{Si}})}({{S}}_{{Si}}-{{S}}_{{Cr}})$$

where *S*_*eff*_ is the effective Seebeck coefficient of the thermocouple; *R*_*Cr-Si*_ is the isolation resistance between the Cr and Si arms that form the Schottky barrier; *R*_*Cr-Ni-S*i_ is the contact resistance between the Cr and Si arms through the Ni ohmic contact; *R*_*Cr*_ is the resistance of the chromium arm; and *R*_*Si*_ is the resistance of the silicon arm. Eq. 1 reveals that the Seebeck coefficient of the thermocouple will reach its maximum and stay very close to the original value of *S*_*Si*_ if we (1) maximize the isolation resistance (*R*_*Cr-Si*_) of the Schottky barrier; (2) minimize the contact resistance (*R*_*Cr*-*Ni-S*i_) of the nickel ohmic contact at the hot point; and (3) minimize the resistance of the chromium and silicon arms (*R*_*Cr*_ and *R*_*Si*_) (see Sections 3 and 4 of SI for more details).

**Figure 2:**
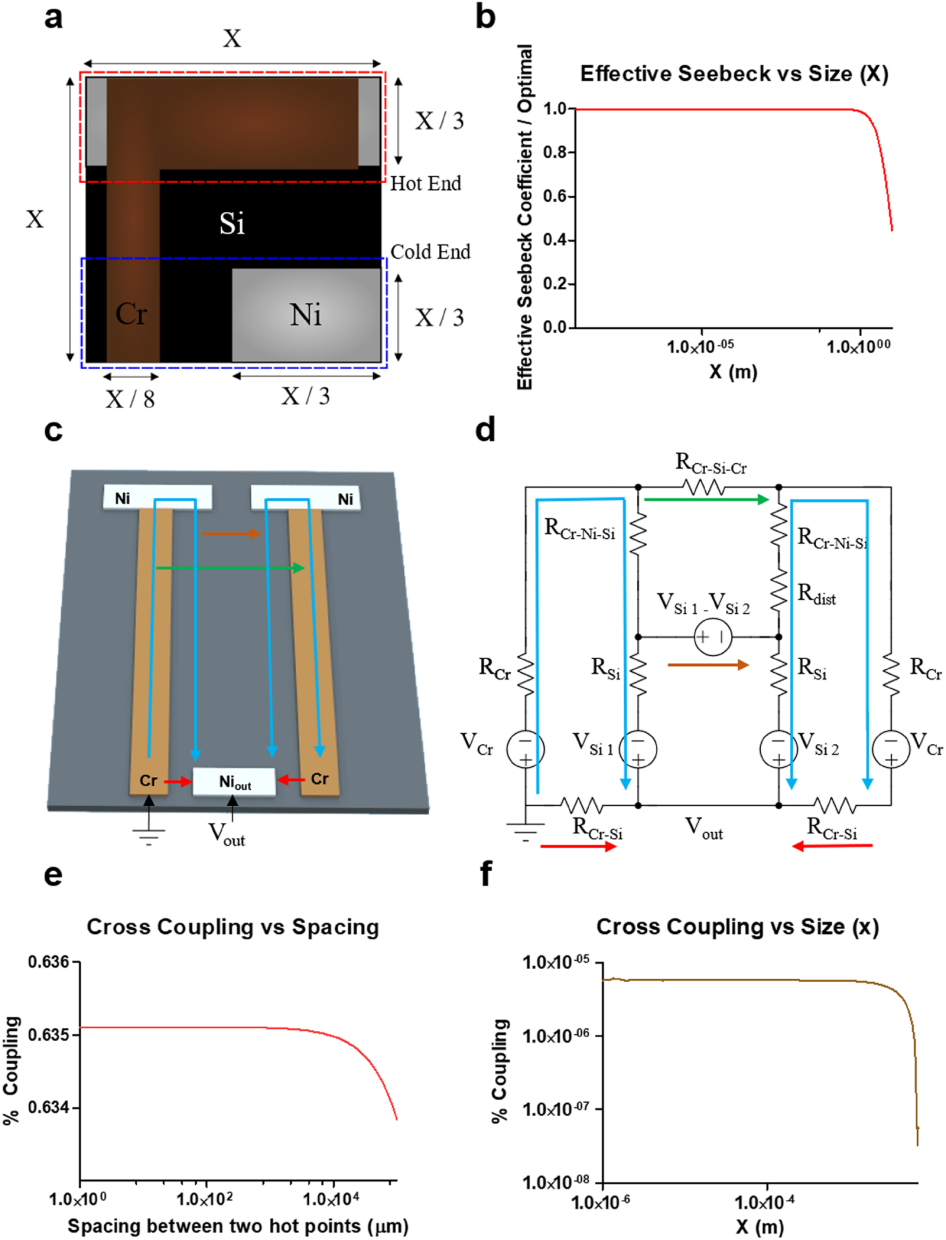
(**a**) Diagram of a thermocouple unit cell which measures the temperature between its hot point (top of the diagram) and the cold end (bottom of the cell). The Cr metal arm is in brown, the Ni ohmic contact in silver, and the silicon substrate in black. In our simulation and analysis, we assume the unit cell has a size of X by X. (**b**) The effective Seebeck coefficient versus the characteristic size (x): the Seebeck coefficient goes down only when the size increases approximately above one meter and remains high and constant for micro scale measurements. (**c**) Schematic of two thermocouples adjacent to each other on a shared silicon substrate with individual current paths. The blue path corresponds to the ideal current path leading to no cross coupling and voltage loss. The red path corresponds to the lossy path through which no thermal voltage is produced. The green arrow indicates one possible cross coupling path where current goes from one Cr strip to the other Cr strip through two Schottky barriers. The brown arrow shows the other cross coupling path where current goes from one Ni hot point to the other through the Si substrate. (**d**) Equivalent circuit model for the two devices shown in (**c**). R_dist_ is an additional resistance that exists between the two points. (**e**) Cross-coupling (%) between the two hot points as a function of physical separation: the measurements represent the percent increase in temperature measured at one cold end caused by the increase of temperature at the hot point of the other device due to electrical crosstalk between them. The magnitude of the coupling remains small and relatively constant (<0.7%) below 1-mm separation. (**f**) Cross-coupling versus the unit size: the cross coupling remains relatively constant and negligible around 1×10^−5^% as the device size is decreased, suggesting that the cross coupling does not limit the device miniaturization.

For our design purpose, we assumed *R*_*Cr-Si*_ to be 700 kΩ in our layout based on geometric calculations and the contact resistivity calculated from the experimentally produced Cr-Si IV curve (Supplemental Figure S2). We sized the areas of the nickel pad and the chrome arm so that *R*_*Cr-Ni-S*i_ and *R*_*Cr*_ stayed below 10 K Ω. This theoretically predicted  that the Seebeck coefficient of the thermocouple would be close to that of silicon (or about 97% of *S*_*Si*_) which would be in the range of 900–1000 µV/K. Further analysis using Eq. 1 also shows that this approach is very lenient on thermocouple miniaturization, and an individual thermocouple can be shrunk down to a few microns in size (Fig. 2(a,b)). Please refer to Section 6 of SI for more details.

A closely packed dense array of the thermocouples can be very useful for high-resolution thermographic imaging but may experience significant inter-device cross-coupling because they share the silicon substrate in common as an electrical-current return path. In order to evaluate the risk of cross-coupling, we expanded the circuit model in Fig. 1(c) to study the interaction between two thermocouples with hot points placed adjacent to each other on a silicon substrate (Fig. 2(c, d)). We varied the spacing between the two devices and performed simulations to find out if a temperature increase at the first hot point would lead to a measured temperature increase due to cross coupling at the second hot point as a function of the distance between the points. The results showed that the cross coupling between the points remains minimal (<1%) down to 1 µm spacing (Fig. 2(e)) as long as we keep the resistance of the Schottky barrier (*R*_*Cr-Si*_) much larger than the resistance of the current-return path in the silicon substrate (*R*_*Si*_). The reason is that, for cross coupling to occur, the current must travel through at least one of the high resistance Schottky barriers (*R*_*Cr-Si*_). Thus, as long as *R*_*Cr-Si*_ is large compared to the other resistances in the circuit, almost no current will flow through the cross-coupling path (Please see Section 5 of SI for more details.) Thus, based on our first-order analysis and computer simulations, it is possible to implement a dense array of thermocouple unit cells, each of which measures 5 µm by 5 µm with an inter-spacing gap of 1 µm using the same materials we utilized in this work. Such high spatial density and resolution promise diverse applications of this thermocouple for macro- or nanoscale 2 dimensional thermography.

### Fabrication and Testing

**Figure 3:**
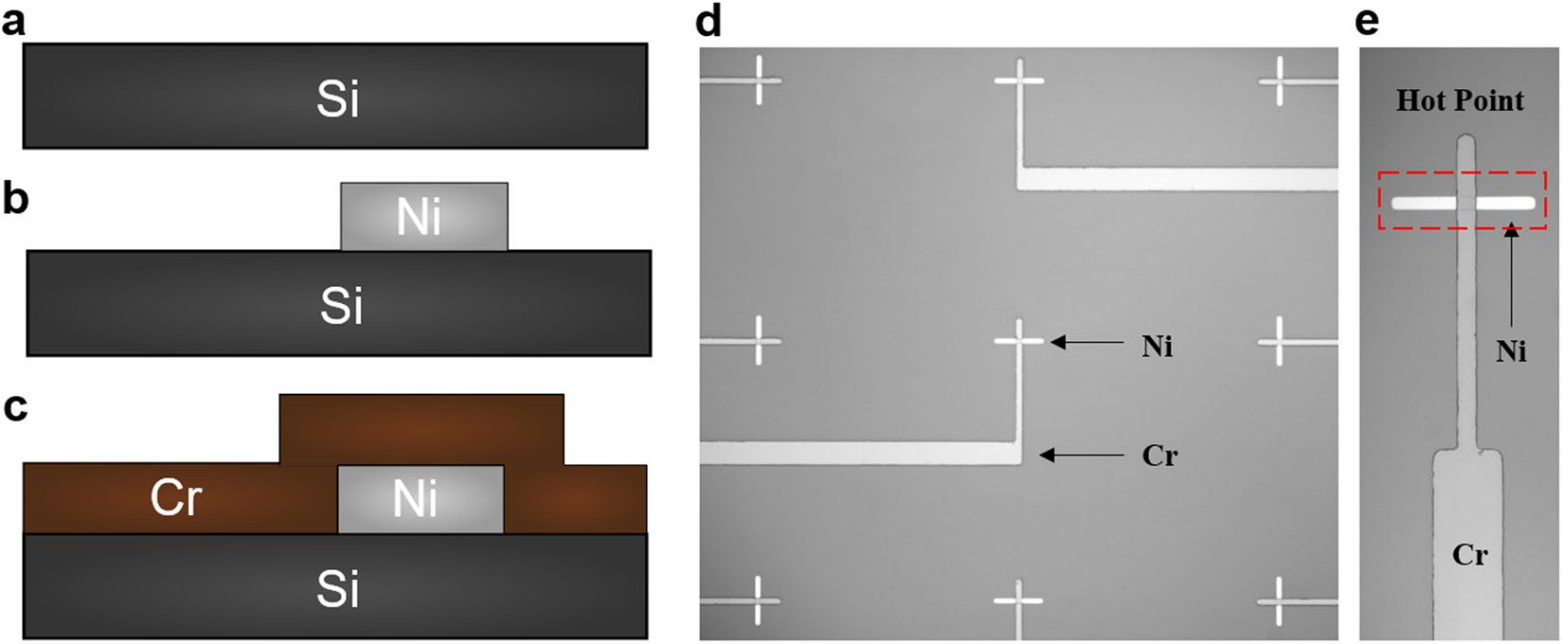
Fabrication of chrome-silicon thermocouples on a silicon wafer. (**a**) Start with a blank silicon wafer (dark grey in the drawing). (**b**) Deposit thin film of nickel (100 nm, silver in the drawing) on the wafer followed by liftoff patterning. (**c**) Deposit and pattern chromium (100 nm, brown in the drawing) on the wafer using liftoff. (**d**) Optical microscope image of an a fabricated 3 × 3 array of thermocouples with nickel patches of different shapes at the hot points. (**e**) A close-up optical microscope image of a hot point: the lighter, horizontal bar is made of nickel.

The fabrication process for these chrome-on-silicon thermocouples is illustrated in Fig. 3, including a photograph of the fabricated devices. The process is comprised of standard photolithography followed by metal liftoff (Fig. 3(a-c)). Each fabricated chip measured 1.5 × 1.5 squared inch and hosted a 3 × 3 array of chrome-on-silicon thermocouples, with each thermocouple junction having dimensions of 20 µm by 20 µm. As discussed in the previous section, our chrome-on-silicon thermocouples can be potentially further miniaturized down to 5 µm sizes (Section 5 in SI 5).

**Figure 4:**
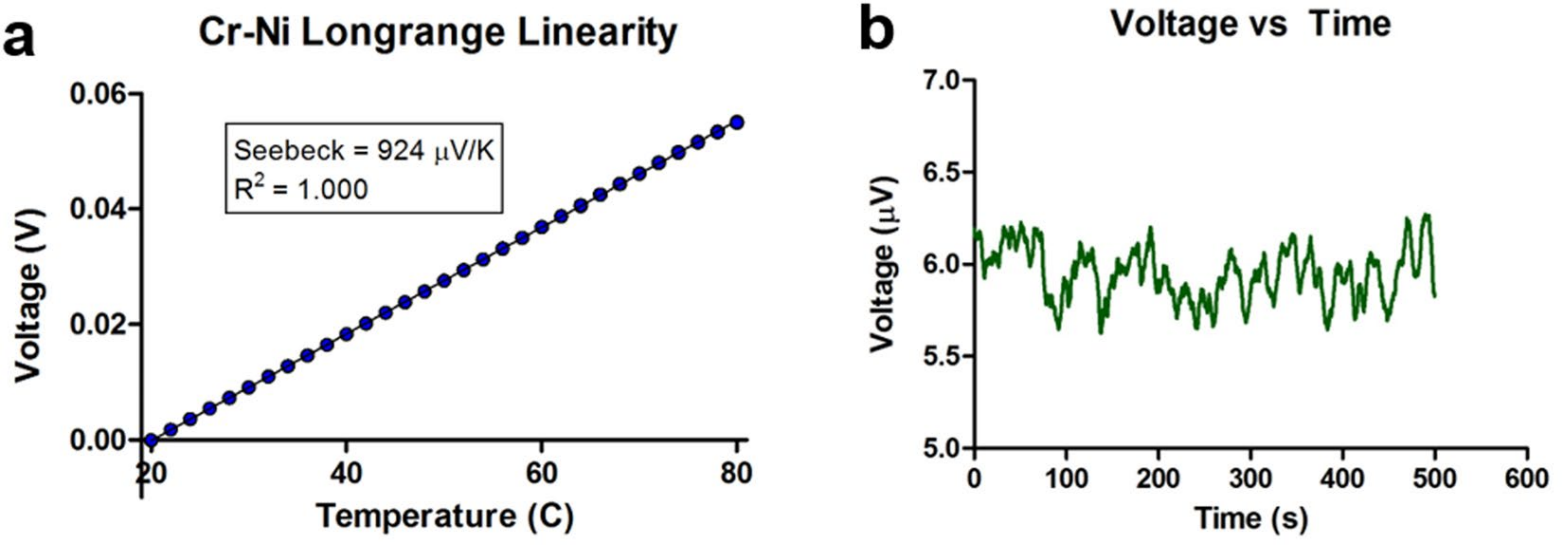
Performance of the chrome-on-silicon thermocouple. (**a**) Thermal voltage vs temperature measurements were made with the cold end constantly held at 20 °C. The device produces linear output between 20 °C and 80 °C. (**b**) Noise measurements - voltage output of the thermocouple over time at a constant temperature. This helps us find the noise floor of the device. The standard deviation of the noise measurements was 0.143 µV.

The performance of the fabricated thermocouples was analyzed over temperatures ranging from 20 °C to 80 °C as shown in Fig. 4(a). The data presented in Fig. 4(a) exhibits an excellent linearity over the tested range of 20 °C to 80 °C with a linearity coefficient of 1.000, ensuring highly accurate and predictable temperature-sensing performance for a wide range of thermal applications. In addition, the Seebeck coefficient of the thermocouples was measured to be 924 µV/K which closely matched values typically observed in lightly p-doped silicon^39,40^. Our thermocouple provides a Seebeck coefficient 30 times larger than that of common Ni-Cr thin film thermocouples, while still being fabricated using straightforward metal-evaporation and liftoff processes. Further measurements and analysis over a wider temperature range showed that the voltage output of the device exhibits nonlinearities as the temperature exceeds 150 °C due to the increased conduction through the Schottky barrier, limiting the max operating temperature of the device to around 150 °C with maximum sensitivity or to 200 °C if nonlinear one-to-one mapping is used between the temperature and voltage (Supplementary Information 7) (Figure S7). This limitation can be easily overcome if a dielectric layer is used to provide electrical isolation between the chrome layer and the silicon substrate.

To analyze the detection limit of the thermocouple, we first calculated the standard deviation of the voltage noise, which was 0.143 µV as shown in Fig. 4(b). We also set the minimum detectable thermal voltage at 0.5 µV (a signal-to-noise ratio > 3 or approximately three times 0.143 µV). Using this information and the measured Seebeck coefficient, we estimate that the thermocouple can detect a temperature change down to 0.5 µV/924 µV/°K or 0.00055 °K in a standard laboratory setting. This is almost two orders of magnitude higher than the previously reported sensitivities of thin film thermocouples, which are shown in Table 1.

**Figure 5:**
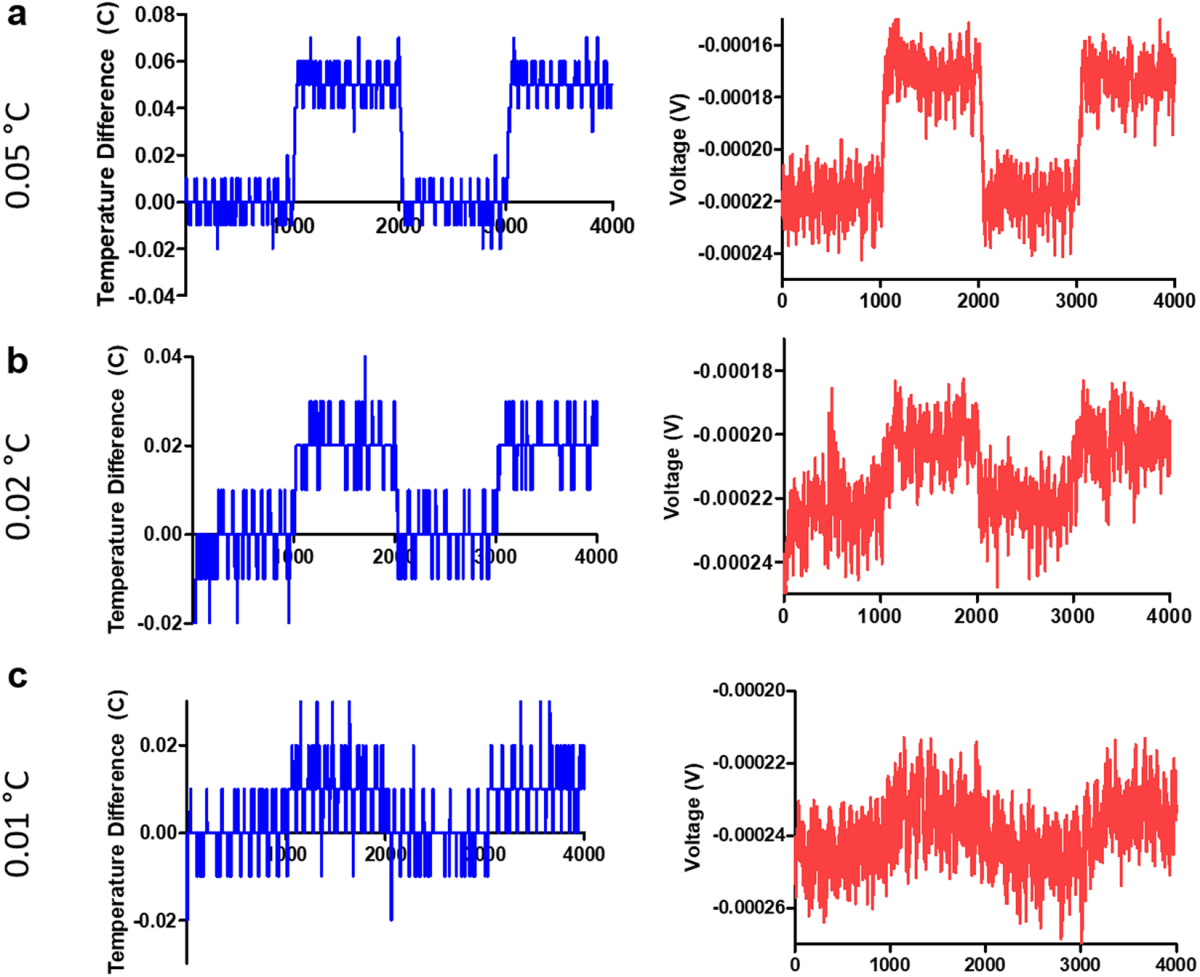
Temperature step measurements. The temperature was raised and lowered by (**a**) 0.05 °C, (**b**) 0.02 °C, and (**c**) 0.01 °C using a hot plate. The reference temperature was obtained using the built-in thermistor of the hot plate (left, in blue) while the voltage was measured on the device (right, in red). The measurements show clear step-shift in voltage level down to the temperature change of 0.01 °C, supporting that 0.01 °C sensitivity can be achieved with this device.

To verify the sensitivity of the thermocouples, a series of temperature measurements were performed by raising the temperature at steps of 0.05, 0.02, or 0.01 °K and measuring the corresponding thermally generated voltage of the device (Fig. 5(a–c)). In all three cases, changes in the measured thermal voltages correspond well with the changes in temperature. We were able to experimentally verify the sensitivity down to 0.01 °K, which was limited by the thermal stability and temperature-sensing resolution of the hotplate setup used in the measurements.

**Figure 6:**
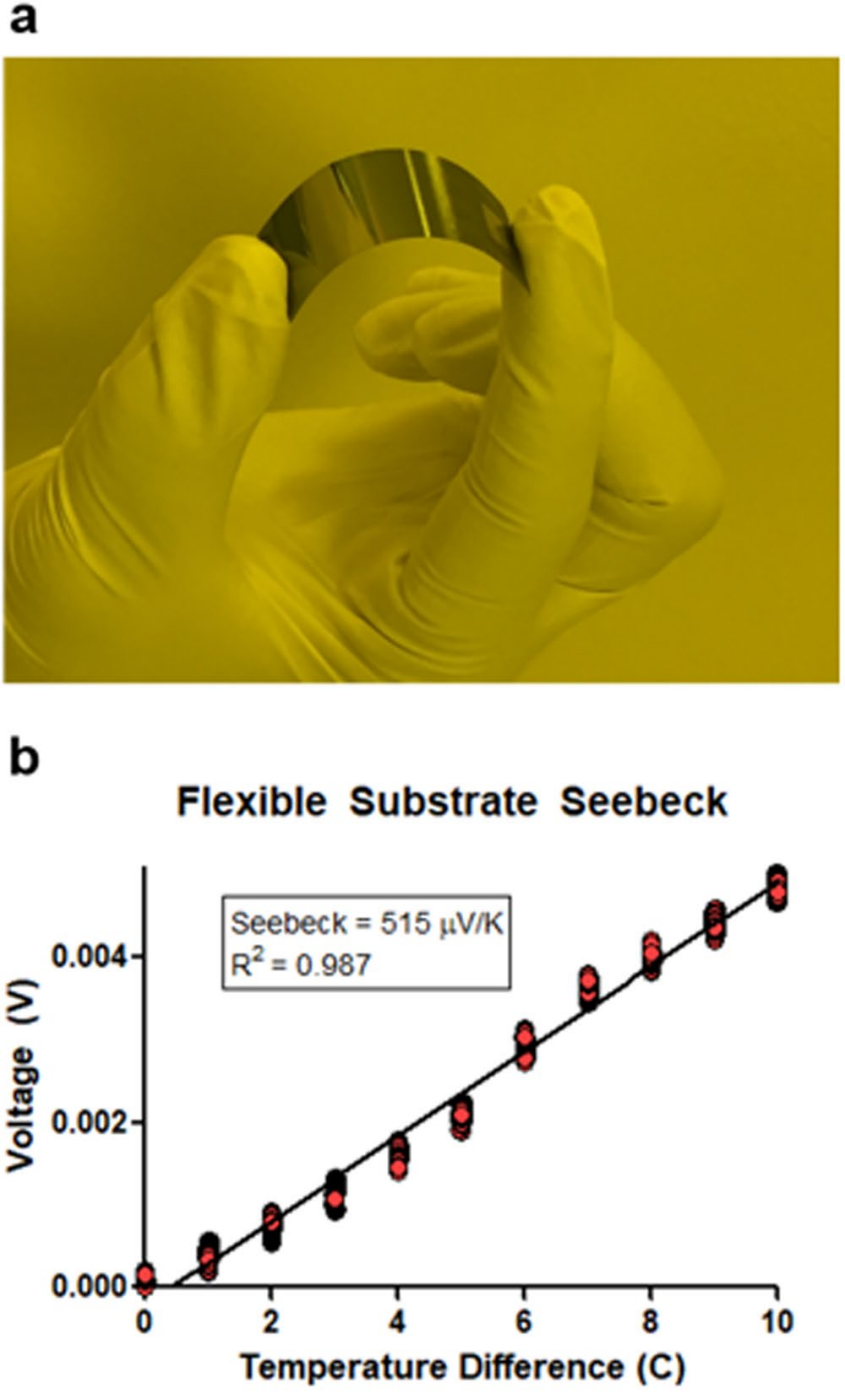
Flexible chrome-on-silicon thermocouple arrays with a high Seebeck coefficient fabricated on a 50  µm-thick double-side-polished silicon wafer. (**a**) In bending testing, the flexible thermocouple array achieved a bending radius of 1.51 cm and a curvature angle of 64.98°. (**b**) Seebeck measurements of the flexible thermocouple array made on a flexible thin wafer – the measurements are highly linear with a high Seebeck coefficient.

Finally, we fabricated chrome-on-silicon thermocouples on thin flexible silicon wafers to demonstrate the use of the thermocouples in more versatile applications including attachable or wearable thermocouples. To obtain flexible silicon wafers with thicknesses around 10–50 µm, we used a strategy widely utilized for the fabrication of flexible electronics^37,38^ and etched double-side-polished silicon wafers down to a thickness of about 50 µm. Then we fabricated the chrome-silicon thermocouples on the thinned wafers using the same processes as before. Thermocouples fabricated on the etched wafers were highly flexible (Fig. 6(a)), allowing repeated bending motions with a bending radius of 1.51 cm and a bending angle around 65 degrees. The devices were flexed at the 65° bending angle repeatedly 50 times with no sign of performance degradation. In addition, the device maintained a high Seebeck coefficient of 515 µV/K (Fig. 6(b)), which was much higher than the values of standard thin-film thermocouples but lower than chrome-silicon thermocouples fabricated on wafers with normal thicknesses. The lower Seebeck coefficient of the flexible thermocouples is attributed to the thinner silicon leading to a larger *R*_*Si*_ which reduces the final Seebeck coefficient of the thermocouples according to Eq. 1. The increase in *R*_*Si*_ that is linearly proportional to the reduction in wafer thickness due to wafer thinning has been plotted in Figure S8.

## Conclusion

We have demonstrated high performance chrome-on-silicon thermocouples using established, simple two-step microfabrication. To our knowledge, this is the first thermocouple design to successfully utilize a silicon substrate as one of the thermocouple arms and its high Seebeck coefficient. The design of our chrome-on-silicon thermocouples was optimized through circuit analysis, and the approach achieves Seebeck coefficients of 924 µV/K and 515 µV/K in rigid and highly flexible forms, respectively. These values far exceed any previously reported standard metal-based thermocouples by a factor of 17–30 and closely match exotic thermocouples made of special alloys. The sensitivity was experimentally demonstrated down to 0.01 °K, which can be further improved toward a theoretical limit of 0.00055 °K. The fabrication process was also kept simple and straightforward by choosing appropriate materials to form electrically isolated chrome and silicon thermocouple arms while using a single metal deposition and lift-off patterning without involving additional fabrication steps. In addition to being implementable in flexible forms, our simulations and first-order analysis suggest chrome-silicon unit cells can be scaled down to 5×5 µm^2^, and adjacent cells can be spaced as close as 1 µm. We expect the design approach and thermocouple platforms demonstrated in this work will be widely utilized in micro-/nanoscale thermometry and thermal mapping specially relevant to plasmonic sensors^11,41–44^, optoelectronics thermal studies and characterizations^45^, and various thermal sensors for biological and physiological analysis^7,8,10^.

## Methods

### Fabrication of thin-film metal thermocouples

Single-side-polished (SSP) (100) silicon wafers were purchased from University Wafer (Boston, USA). These included p-type silicon wafers doped with Boron (resistivity: 1–30 Ω cm), n-type wafers doped with Phosphorus (resistivity: 1–10 Ω cm), and undoped wafers (resistivity: >10000 Ω cm). Photolithography and metal-liftoff were used to fabricate the thin-film thermocouples on the wafers (Fig. 3). AZ5214 photoresist was spin-coated onto the wafers and then patterned using a Suss Microtech MA6/BA6 mask aligner. Nickel (Ni, thickness: 100 nm) was then deposited onto the sample using electron-beam (e-beam) evaporation and acetone was used to lift-off the metal. AZ5214 photoresist was spin-coated on the wafers again and patterned using the mask aligner and AZ 400K photoresist developer. Finally, a 100-nm layer of Cr was deposited onto the samples using e-beam evaporation followed by a lift-off process in acetone.

### Substrate Tests

To see the difference in the measured Seebeck coefficient of Ni on differently doped substrates (Supplemental Information Section 2), Ni strips with widths of about 500 microns and lengths of about 3 cm were fabricated onto a p-doped (1–30 Ω cm), n-doped (1–10 Ω cm), and undoped wafers using photolithography and metal-liftoff. Electrodes were placed on either end of the strips and their Seebeck coefficient was measured as discussed in the results.

### Flexible thermocouples

Double-side-polished (DSP) p-doped silicon wafers were obtained from Ultrasil Corp. (Hayward, CA, USA) doped with Boron (resistivity: 1–30 Ω cm). Ni-Cr thin film thermocouples were fabricated on these wafers as described earlier. The wafers were then thinned down to a thickness of about 50 µm using deep reactive ion etching (DRIE). Santovac-5 high-vacuum fluid (SantoLubes LLC, SC, USA) was gently spread over the device side of the wafers as a protective layer before the etching process. After the etch, the sample was placed in a PG remover to remove the high-vacuum fluid. The thinned 50- µm-thick chips were then cleaned with DI water, dried with N_2_-gun, and tested.

### Current-voltage (IV) measurements

For current-voltage measurements, an ammeter was put in series between the sample and a voltage source. The voltage on the voltage source was initially set at 0 V and then was slowly increased. The voltage on the voltage source and the current from the ammeter were recorded.

### Measurement setup

Copper wires were attached onto the metal pads on the wafer using thermal silver epoxy (8331S-15G, MG Chemicals). The hot and cold ends of the thermocouple were attached to two separate hot plates (CP-036HT, TE Technology). Aluminum foil was placed between the sample and the hot plates to protect the measurement from the plate-generated electrical noise. A thermistor was placed on each plate to provide the reference temperature (MP-2444, TE Technology). The thermistor was epoxied into place using an electrically insulating thermal epoxy to ensure good thermal contact (8329TCM-6ML, MG Chemicals). Each plate regulates its temperature within ± 0.01 °C of the target temperature (TC-720, TE Tech). The temperature was varied depending on the measurement being taken (see different measurements below), and the resulting voltage across the device was recorded using a nanovoltmeter (2182 A, Keithley) at a rate of approximately 0.5 s per measurement.

### Seebeck coefficient measurement

Initially the temperature plates were both set at 30 °C. Then 1000 successive measurements were taken on the voltmeter and the difference in the temperature of the two plates. The temperature of the cold side was maintained at 30 °C while the temperature of the second temperature plate (hot side) was raised by 1 °C. The measurements were repeated, and the process was continued until temperature of the hot side reached 40 °C.

### Working range measurement

In order to test the performance of the thermocouple over a wider range of temperatures, both temperature plates were set to 20 °C initially. Then 1000 successive measurements were taken on the voltmeter. The temperature of the plate on the hot side was then raised by 2 °C, and the measurements were repeated. This process was repeated until temperature of the hot side reached 80 °C.

### Sensitivity measurements

The temperature of the plates was initially set to 30 °C and 1000 measurements were taken on the voltmeter. The temperature on the hot side was then raised by 0.05 °C, 0.02 °C, or 0.01 °C. 1000 measurements were then taken again, and the temperature on the hot side was returned to the base value.

### Device Noise Measurement

To measure the noise of the device, the voltage of the device was continuously measured by the voltmeter over a 500 s period while keeping the device at room temperature and inside electrical shielding.

### Bending Radius Measurement

To calculate the bending radius images of the bent device were taken. The images were analyzed using ImageJ to measure the chord and the rise, and from those the bending radius was calculated.

### Data analysis

We used Matlab R2016b (The Mathworks, Inc.) and GraphPad Prism 5.01 (GraphPad Software, Inc.) to analyze the data and create graphs.

## Electronic supplementary material

Supplementary Information

## Data Availability

The data generated during and/or analyzed during the current study are included in this manuscript/supplementary information file and are further available from the corresponding author on reasonable request.
