# Supplementary material for: High-performance flexible metal-on-silicon thermocouple
Source: Sci Rep. 2018 Sep 13;8:13725. doi: 10.1038/s41598-018-32169-9 (PMC6137040; doi:10.1038/s41598-018-32169-9)
Supplement: Supplementary file 1 — Supplementary Information [file 41598_2018_32169_MOESM1_ESM.pdf]

**Supporting Information**

# High-performance flexible metal-on-silicon thermocouple

*Daniel Assumpcao,[1] Shailabh Kumar,[2] Vinayak Narasimhan,[2] Jongho Lee, Hyuck Choo*[,1,2]*

[1]Department of Electrical Engineering, [2]Department of Medical Engineering,

California Institute of Technology, Pasadena, CA 91125, United States

School of Mechanical Engineering,

Gwangju Institute of Science and Technology, Gwangju, Republic of Korea

Corresponding author *E-mail: hchoo@caltech.edu

**1. Thermocouple Design: Schottky-Barrier and Ohmic-Contact Formations on Silicon**

The Seebeck effect relies on difference in energy of charge carriers at the hot end of a material as compared to the cold end. Charge carriers at the hot end tend to have higher thermal energy leading to an increased diffusion of charge towards the cold end (Figure S1(a)). The resulting imbalance leads to generation of an electric potential between the two ends, whose magnitude is directly proportional to the Seebeck coefficient ($S$) of the material. A thermocouple uses a pair of materials with different $S$ values, so that a potential difference is generated between the two different materials at the junction and used to estimate the difference in temperature between the cold end (usually externally controlled as a reference) and the hot end.

We consider a thin metal film and a p-type silicon substrate. If the two materials are initially isolated, then application of a temperature gradient across this device should result in generation of two thermoelectric voltages by the Seebeck effect, one in the silicon substrate and the other in the metal. The voltage generated in the silicon will be much larger than the voltage generated in the metal since silicon's Seebeck coefficient ($S_{Si}$) is much larger than the metal's Seebeck coefficient ($S_M$) (Figure S1(a)). In the proposed approach, the current conducts through the substantially thick silicon substrate (or a large cross-section) and experiences a very low level of resistive loss, which results in a large measurable thermal voltage.

Next, we consider that the thin metal film is in contact with the p-type silicon substrate. Whether charge can conduct between a semiconductor and a metal depends on the metal's work function. If the metal has a work function less than that of the p-type silicon, a depletion layer will form at their interface called a Schottky barrier (Figure S1(b)) [1]. Charge will not flow across this barrier normally, leaving the thermally generated charge carrier densities and voltages unchanged

(Figure S1(c)). However, if the work function is close to or larger than that of the p-type silicon there will be no depletion region and charge can freely conduct across the interface, called an ohmic contact (Figure S1(d))[1]. Now when a temperature gradient is applied, the two charge gradients in the two materials will diffuse together as the substrate itself acts as an arm in parallel to the metal arm (Figure S1(e)). Since the semiconductor initially had a much larger charge carrier concentration gradient, larger current will diffuse into the metal. This leads to a larger generated potential measured across the metal, implying the Seebeck coefficient of the semiconductor is properly utilized. Thus, two metal arms can be deposited on a shared silicon substrate, (one with a work function larger than that of silicon, called the coupling arm since it couples to silicon's Seebeck coefficient, and one with a work function less than that of the silicon, called the isolation arm since it is isolated from the silicon), and can be connected to form a metal-on-silicon thermocouple. The overall Seebeck coefficient of this chrome-on-silicon thermocouple will be the difference between the Seebeck coefficients of the two arms, optimally $S_{Si}$ - $S_M$, which will be large due to a high value of $S_{Si}$. Thus, through properly selecting fermi levels of the materials, ohmic and Schottky contacts can be selectively formed, creating metal-on-silicon thermocouples with large Seebeck coefficients.

To experimentally verify the formation of the desired Schottky barrier and ohmic contacts, chips were fabricated by depositing Ni and Cr on p-doped Si wafers. The work function of the p-type silicon wafers was calculated to be 5 eV from the doping level[1]. Ni (workfunction of 5.15 eV, higher than silicon) and Cr (workfunction of 4.5 eV, lower than silicon) were expected to form an ohmic contact and a rectifying Schottky-barrier contact, respectively[2]. The formation of an ohmic contact between a metal and p-doped semiconductor requires that the work function of the metal be close to or larger than the work function of the semiconductor[1]. A cross-sectional representation

of the chips can be seen in Supplementary Figure S2 (a, b). The IV curves of these two samples were measured as shown in Figure S2(c, d). The IV curve of the Ni was linear, indicating formation of an ohmic contact with the substrate which has a low resistance, and this easily allows transfer of electrons between the semiconductor and the metal[3, 4]. However, the IV curve of the Cr had a rectifying shape as seen in Figure S2(d)[4], indicating the formation of a Schottky barrier.

**2. Tapping into the Seebeck coefficient of the silicon substrate: a single ohmic contact case**

To tap into the large Seebeck coefficient of the silicon substrate, standalone Ni pads were fabricated on lightly p-doped, n-doped, and undoped Si substrates and the Seebeck coefficient was characterized (Figure S3(a)). Formation of an ohmic contact between Ni and Si was verified using IV measurements (Figure S2). Seebeck coefficients were measured across each individual single Ni ohmic contact pad ($S_{Ni}$) on a p-doped substrate, and they averaged 412 μV/K. On the other hand, Ni pads on n-doped and undoped substrates formed a Schottky barrier, and $S_{Ni}$ remained around -10 μV/K, close to the bulk Seebeck coefficient for Ni [5]. Thus, we experimentally verified the formation of an ohmic contact between Ni and Si led to increase in the measured Seebeck coefficient due to the contribution of the Si substrate, as postulated earlier.

To further analyze the above effect, a circuit model was created for a metal strip on a silicon substrate. The circuit model considers two voltage sources, which are the two Seebeck voltages generated in the metal and silicon substrate separately, with the source resistance in parallel with each other as shown in Figure S4(a). Solving the circuit generates a formula for the equivalent Seebeck coefficient of a thermocouple arm with two sources in parallel ($S_{arm}$).

$$S_{arm} = \frac{S_{Si}R_M + S_M(R_{ohm} + R_{Si})}{R_M + (R_{ohm} + R_{Si})} \quad \text{(S1)}$$

Where $R_M$ is the resistance of the metal, $R_{Si}$ is the resistance of the silicon substrate, $R_{ohm}$ is the resistance between the substrate and the metal strip, $S_{Si}$ and $S_M$ are the Seebeck coefficients of the silicon and the metal, respectively.

In the case where an ohmic contact is formed between the p-doped substrate and the metal, $R_{ohm}$ can be considered much smaller compared to $R_{Si}$ and $R_M$. As a result, Equation S1 can be rewritten as:

$$S_{arm} \approx \frac{S_{Si}R_M + S_M R_{Si}}{R_M + R_{Si}} \qquad (S2)$$

Silicon has a larger Seebeck coefficient than metals so $S_{Si} > S_M$. As the metal films have thin, narrow, and long dimensions, their resistance ends up higher than that of the silicon wafers, such that $R_{Si} < R_M$. Hence equation (S2) indicates that $S_{arm}$ approaches $S_{Si}$ under these conditions. We measured the resistances in the devices: $R_{Si} \approx 500\ \Omega$, $R_M \approx 1\text{k}\Omega$, and $R_{ohm} \approx 100\ \Omega$. Using the known Seebeck coefficients $S_{Si} \approx 1000$ µV/K and $S_M \approx -10$ µV/K [5, 6], it can be calculated that $S_{eff} \approx 600$ µV/K, indicating a huge increase due to the ohmic contact. Intuitively this can be understood as a decrease in the metal-silicon interface resistance leading to an increase of current through the silicon substrate arm, leading to silicon contributing a larger portion of the Seebeck coefficient. On the undoped or n-doped sample, $R_{ohm}$ is very large due to the formation of the Schottky barrier, hence $S_{arm}$ is approximately equal to $S_M$, which is much smaller than $S_{Si}$, further verifying the substrate test results.

To provide further verification for equation S1, identical thin Ni strips were patterned on Si chips (approximately 4 cm by 1.5 cm) whose widths were varied to change the bulk substrate resistances ($R_{Si}$). The resistance and Seebeck coefficients of the devices were measured, and $R_{Si}$ was calculated

using the overall resistance and the resistance of the same Ni pattern as deposited on an undoped Si substrate. The plot of the overall resistance (the combination $R_{Si}$ and $R_M$) versus the measured Seebeck coefficient on the Ni is shown in Figure S3(b). The data shows good agreement with the expected curve calculated from Equation S1.

**3. Formation of a silicon thermocouple arm through two Ni-Si ohmic contacts**

We extended the approach to create a silicon arm (of a thermocouple) with a very high Seebeck coefficient. As stated before, a thermocouple consists of two arms (Cr and silicon through Ni in this case), and in order to maximize the overall effective Seebeck coefficient $(S_{eff})$, the difference in Seebeck coefficients of the two arms must be maximized i.e. $S_{eff} = S_{arm}(Si\ through\ Ni) - S_{arm}(Cr)$, where $S_{\mathrm{eff}}$ is the final Seebeck coefficient that results from combining the two thermocouple arms, and hence we need to maximize $S_{arm}(Si\ through\ Ni)$. In our analysis, this Seebeck coefficient is found to be a function of the electrical resistances as described in Eq. S1, and as a result, $S_{eff}$ can be maximized by optimizing the values of the resistances. We aim at $S_{eff} \approx S_{\mathrm{Si}}$.

Equation S1 shows that $R_{ohm}$ and $R_{Ni}$ (the resistance of the metal pad or strip, with Ni being metal in this case) approaching zero and infinity, respectively, are optimal conditions for maximizing $S_{arm}(Si\ through\ Ni)$. Formation of an ohmic contact using Ni on p-doped silicon substrate ensures that $R_{ohm}$ is very small compared to the other resistances in the circuit. In order to increase $R_{Ni}$ to infinity, the circuit should be left open, and this would translate to having a discontinued Ni strip or two physically separated Ni pads as shown in Figure 1(b), which makes $R_{Ni}$ infinite. This implies that the metal (Ni) should not provide any electrical connection between the hot and cold ends, and it just serves as an ohmic contact at each end. Having two physically separated Ni ohmic contacts, one at the hot end and the other at the cold end, makes all the current

flow through the Si substrate, and the Si substrate becomes one of the two arms that form a thermocouple. This causes the Seebeck voltage to be dominated by the Si substrate, resulting in $S_{arm} \approx S_{Si} \approx 900$ μV/K (for lightly p-doped Si), about twice the measured Seebeck coefficient of single Ni ohmic contacts formed on p-type Si substrate discussed in the previous section.

To keep the coefficient of the metal arm low, we must effectively isolate it from the silicon substrate i.e. $S_{arm} \approx S_{Cr}$.. To ensure this, $R_{ohm}$ must be made very large. For our substrate, this is achieved by choosing a metal such as chrome that forms an electrically blocking Schottky barrier with the p-doped silicon substrate. Thus by taking into account the above optimizations, the optimal Cr-on-Si thermocouple will look like Figure 1(b) where Si and Cr serve as two arms of the thermocouple, and Ni serves as a coupling component between the chrome and the silicon.

**4. Circuit analysis of a complete thermocouple with the two arms**

Figure 1(b) shows the final design that combines the optimized Seebeck coefficients of both thermocouple arms to achieve the best engineered performance of the thermocouple. An equivalent circuit for the whole device can be seen in Figure S4(b). In the proposed thermocouple, the current can flow through two paths: it can either go around the entire thermocouple as intended (through the thermally isolated metallic arm, then through the coupled metallic arm into the silicon substrate), leading it to encounter the Cr arm resistance $R_{Cr}$, the silicon resistance $R_{Si}$ and the contact resistance between the metal arm and silicon arm through the ohmic contact $R_{Cr\text{-}Ni\text{-}Si}$, but also picking up the thermoelectric voltages; or it can cut through the barrier between the isolated arm and the substrate encountering a resistance $R_{Cr\text{-}Si}$ and contributing a voltage of 0 V. Solving the equivalent circuit generates the following equation for the measured Seebeck coefficient:

$$S_{eff} = \frac{R_{Cr-Si}}{R_{Cr-Si} + (R_{Cr} + R_{Cr-Ni-Si} + R_{Si})} (S_{Si} - S_{Cr}) \qquad (S3)$$

In order to further analyze this equation, an expression must be obtained for $R_{Cr-Si}$. The isolation resistance is from the Schottky barrier between the isolation arm and the substrate. The current across a Schottky barrier is given by the equation[7]:

$$I = AA^{*}T^{2}e^{\frac{-q\phi_B}{kT}}\left(e^{\frac{qV_a}{kT}} - 1\right) \qquad (S4)$$

Where A is the area of the device, $A^*$ is Richardson's constant, $\phi_B$ is the potential barrier height, T is the temperature, and $V_a$ is the applied voltage. Since the thermoelectric voltages  generated by thermocouples are small throughout the material, the small-signal-equivalent resistance value assuming $V_a$ ~ 0 V can be used:

$$R_{Cr-Si} = \frac{dV_a}{dI} = \frac{1}{\frac{q}{kT}AA^{*}T^{2}e^{\frac{-q\phi_B}{kT}}}$$

$$= \frac{ke^{\frac{q\phi_B}{kT}}}{AA^{*}TKq} \qquad (S5)$$

Thus, by choosing a metal that forms a large potential barrier to silicon that increases $\phi_B$, this resistance value $R_{Cr\text{-}Si}$ can be made large, electrically isolating the arm from the silicon substrate.

From equation 1, the optimization of the measured effective Seebeck coefficient ($S_{eff}$) requires maximization of $R_{Cr\text{-}Si}$, the resistance between the isolation trace and the silicon substrate, whereas the other resistances must be minimized. If the width of the isolated metallic arm becomes very thin, $R_{Cr}$ will increase so that $R_{Cr}$ >> ($R_{Cr\text{-}Ni\text{-}Si}$ + $R_{Si}$). This means that $S_{eff}$ is approximately equal to:

$$S_{eff} \approx \frac{R_{Cr-Si}}{R_{Cr-Si} + R_{Cr}}(S_{Si} - S_{Cr}) \qquad (S6)$$

Assuming that the isolation has a uniform width w, length l, and thickness t then from the resistance formula $R_M$:

$$R_{Cr} = \rho_{Cr} \frac{l}{t \cdot w} \qquad (S7)$$

Since $A = l \cdot w$, implying $R_{iso} \propto (wl)^{-1}$ thus using a proportionality constant α:

$$R_{Cr-Si} = \frac{\alpha}{wl} \qquad (S8)$$

Thus, the overall actual Seebeck coefficient in this limiting case is:

$$S_{eff} \approx \frac{\alpha/(l \cdot w)}{\alpha/(l \cdot w) + (\rho_{Cr} \cdot l)/(t \cdot w)} (S_{Si} - S_{Cr})$$
$$= \frac{\alpha t}{\alpha t + p_{Cr} l^2} (S_{Si} - S_{Cr}) \qquad (S9)$$

The final Seebeck coefficient of the device is no longer a function of the width of the metallic isolation arm. Thus, the effectiveness of the device does not degrade with a smaller metallic contact, indicating that the measurement point can be made as small as necessary for micro and nanothermography. This analysis shows the ease of scalability of this principle for large- or small-scale thermography using simply fabricable thin-film thermocouple devices, and this scalability is studied in more detail in SI 6.

**5. Multi thermocouple simulation**

The scope and influence of having multiple measurement points on the substrate were analyzed. The risk of inter-point coupling where temperature change on one point can lead to a

measured temperature difference with a nearby measurement point was considered. An equivalent circuit model for two points on a shared substrate was developed and is shown in Figure 2(c, d). In order to analyze this circuit, the Simulation Program with Integrated Circuit Emphasis (SPICE) was used. The different resistances in the circuit were varied and the output Seebeck coefficient was measured

The results of the simulation are shown in Figure S5. The overall Seebeck coefficient drops as $R_{Cr.}$ $R_{Si}$, and $R_{Cr\text{-}Ni\text{-}Si}$ increase relative to $R_{Cr\text{-}Ni}$, indicating a need to keep the Cr arm's resistance low, the silicon's resistance low, or increasing the isolation resistance of the Schottky barrier. This condition is similar to substrates with a single measurement point, solved analytically previously in equation (S3). Figure S5(b) shows the percent of cross coupling between two points, calculated by considering the increased temperature at one point after a 1 degree temperature increase at the second point due to electrical interference . This analysis indicates that interpoint coupling can be restrictive towards multipoint measurements if $R_{Si}$ is larger than $R_{Cr\text{-}Si}$. Thus, as long as steps are taken to ensure $R_{Cr\text{-}Si}$ is large by choosing a material for the isolation arm that forms a large Schottky barrier with the substrate, this can be avoided. $R_{Cr\text{-}Si}$ can be estimated using equation (4), assuming $A \approx 10^{-4}$ $m^2$ (calculated from pattern used in the final devices below), $A^* = 32$ A $cm^{-2}$ $K^{-2}$ [7], T = 303K, and $\phi_B$ = 0.8V. This yields $R_{Cr\text{-}Si} \approx 700$ kΩ. This is much larger than $R_{Si,}$ which was measured to be around 100Ω in the 500μm lightly p-doped silicon wafers used, indicating ideal behavior. Since in most cases the device can be designed such that $R_{Si}$ is less than $R_{Cr\text{-}Si}$, multiple hot points can be placed on the same silicon substrate enabling measurement of temperature over an array of points and thermal mapping.

**6. Unit Cell Miniaturization**

A silicon thermocouple unit cell was analyzed to determine how miniaturization could impact the performance. A unit cell with characteristic size $x$ is shown in Figure 2(a). The hot junction is at the top of the diagram, at the intersection of the Cr (brown) and the Ni (silver). The two contacts where the potential is measured across is at the bottom of the diagram. The equivalent circuit for this is given by Figure S4b) and the effective Seebeck coefficient is given by equation S3. The four characteristic resistances are thus estimated by the following four equations.

$$R_{Cr} = \rho_{Cr}\left(\frac{x}{\frac{x}{8}\cdot\tau_{Cr}}\right) = \rho_{Cr}\frac{8}{\tau_{Cr}} \qquad (S10)$$

$$R_{Si} = \rho_{Si}\left(\frac{x}{x\cdot\tau_{Si}}\right) = \rho_{Si}\frac{1}{\tau_{Si}} \qquad (S11)$$

$$R_{Si-Ni-Cr} = \frac{\rho_{Si-Ni-Cr}}{x\cdot\frac{x}{3}} + \frac{\rho_{Si-Ni-Cr}}{\frac{x}{2}\cdot\frac{x}{3}} = \frac{9\rho_{Si-Ni-Cr}}{x^2} \qquad (S12)$$

$$R_{Si-Cr} = \frac{\alpha}{x\cdot\frac{x}{8}} = \alpha\frac{8}{x^2} \qquad (S13)$$

Where $\rho_{Cr}$ is the resistivity of the isolation metal (Cr), $\rho_{Si}$ is the resistivity of the silicon, $\rho_{Si-Ni-Cr}$is the contact resistivity between the ohmic contact and the silicon substrate, $\alpha$ is the effective contact resistivity between the Cr arm and the silicon substrate (see SI 3), $\tau_{Cr}$ is the thickness of the Cr arm, $\tau_{Si}$ is the thickness of the silicon, and x is the characteristic length. Given these the effective Seebeck coefficient of the unit cells becomes:

$$S_{eff} = \frac{R_{Cr-Si}}{R_{Cr-Si} + (R_{Cr} + R_{Cr-Ni-Si} + R_{Si})}(S_{Si} - S_{Cr})$$

$$= \frac{8\alpha}{x^2\left(\frac{8\rho_{Cr}}{\tau_{Cr}} + \frac{\rho_{Si}}{\tau_{Si}}\right) + 9\rho_{Cr-Ni-Si} + 8\alpha}(S_{Si} - S_{Cr}) \qquad (S14)$$

For numerical analysis, the following values were used for the parameters: $\rho_{Cr} = 10^{-6}\ \Omega \cdot m$, $\rho_{Si} = 10^{-2}\Omega \cdot m$, $\rho_{Cr-Ni-Si} = 10^{-4}\Omega \cdot m^2$, $\alpha = 10^3\Omega \cdot m^2$, $\tau_{Cr} = 100\ nm$, $\tau_{Si} = 500\ \mu m$.

The effective Seebeck coefficient is plotted versus the unit cell length in Figure 2(b). Thus, the effective Seebeck coefficient improves as the dimensions are miniaturized. This is due to the fact that the resistance of the Schottky barrier ($R_{Si\text{-}Cr}$) increases as the unit cell length is decreased whereas the resistance of the chromium arm ($R_{Cr}$) and silicon arm ($R_{Si}$) remain constant. From Equation S3 an increased Schottky barrier resistance as compared to the chromium and silicon arm resistance leads to an increased effective Seebeck coefficient. Since the contact resistance between the silicon and chromium arms ($R_{Si\text{-}Ni\text{-}Cr}$) also increases as the unit cell length is decreased with the same rate of increase as the Schottky barrier resistance, the effective Seebeck coefficient will approach a limit as the unit cell length approaches 0. Thus, the decrease in the effective Seebeck coefficient is not an issue with miniaturization.

Another consideration for miniaturization is it leading to increased inter point cross coupling. To evaluate this the inter point coupling was simulated using the same model as in Supplemental Information Section 5. The inter point coupling versus the unit cell size is displayed in Figure 2(f). As the device becomes smaller, the inter-point coupling saturates and does not significantly increase. This is again due to the scaling of the involved resistances.

Another potential issue is that decreasing the dimensions of the device can lead to a higher resistance. A higher resistance leads to higher Johnson noise and if the resistance becomes too high it will not be measurable on a voltmeter. Calculating the resistance of the circuit in Figure S4(a):

$$R_{out} = \frac{R_{Cr-Si} \cdot (R_{Cr} + R_{Cr-Ni-Si} + R_{Si})}{R_{Cr-Si} + (R_{Cr} + R_{Cr-Ni-Si} + R_{Si})}$$

$$= \frac{8\alpha[x^2\left(\frac{8\rho_{Cr}}{\tau_{Cr}} + \frac{\rho_{Si}}{\tau_{Si}}\right) + 9\rho_{Cr-Ni-Si}]}{x^2[x^2\left(\frac{8\rho_{Cr}}{\tau_{Cr}} + \frac{\rho_{Si}}{\tau_{Si}}\right) + 9\rho_{Cr-Ni-Si} + 8\alpha]} \qquad (S15)$$

The resistance versus length is plotted in figure S6(a). The overall resistance increases as the device is scaled down. Assuming the maximum resistance of a voltage source that can still be measured by a voltmeter is 10MΩ, the minimum length is calculated using the above formula is around 5 µm. Thus, this provides a minimum dimensions of a unit cell for a selectively-conduction based silicon thermocouple with the design in figure 2(a).

Finally, the effect of the increased resistance on worsening the device sensitivity was analyzed. An increased resistance causes an increase in the Johnson-Nyquist electrical noise, thus impacting the ideal sensitivity of the device which could further limit the practical size of the device. In order to determine the optimal sensitivity of the device (assuming only Johnson-Nyquist noise) at any given size the Johnson-Nyquist noise was calculated for a given resistance and divided by the Seebeck coefficient to determine the thermal sensitivity. The results are shown in Figure S6(b). As seen from the graph, although the sensitivity of the device becomes worse as the length shrinks, the optimal sensitivity is still very low even down to the micron range (about 1E-5 °K), and any real device would likely be limited by other larger sources of noise instead of the Johnson-Nyquist noise at that scale. Thus, the only significant limit due to miniaturization is due to the resistance getting too high as explained in the previous paragraph. Therefore, this basic analysis shows the device can miniaturized down to the single micron scale.

## 7. Long range nonlinearities

Although we are primarily concerned with having high sensitivity and linearity in the 20 to 80 °C range in the metal-on-silicon thermocouples due to the focus on biological application,

it is possible that at higher temperatures the voltage output of the device may become nonlinear. Our device in particular is sensitive to higher temperature nonlinearities due to the use of the Schottky barrier as the isolation between the substrate and the metal leg. At higher temperatures, charge carriers have enough thermal energy to bypass this barrier, causing the resistance of the Schottky barrier to drop significantly. This drop in isolation resistance at higher temperatures leads to a drop in the effective Seebeck coefficient, thus causing a nonlinearity.

To measure this potential nonlinearity, a different measurement system had to be used than before since the temperature of the previously used hot plate was limited to 100 °C. In this new system, the cold end of the thermocouple was still attached to one hot plate at a cold temperature but now a soldering gun was hung above the hot end of the device and its temperature was controlled. This allowed for temperatures above 100 °C to be applied to the thermocouple, at the expense of much more fluctuations in the temperature and thus noisier results.

The measured output voltage versus the temperature of the soldering gun is shown in figure S7(a). As shown, the voltage output becomes extremely nonlinear as the temperature of the hot end approaches 200 °C, thus limiting the maximum temperature in the maximum sensitivity and linear regime of the metal-on-silicon thermocouples to about 150 °C. However, it should be noted that through using one to one nonlinear temperature and voltage mapping this temperature range can be extended up until 200 °C.

To better understand the results and ensure that the effects described earlier are the reasons behind it, circuit simulations were performed. To capture this effect, the Schottky barrier could no longer be approximated as a resistor as before, and instead it had to be modeled as a diode. The circuit in Figure S7(b) was used as a model, which consisted of just replacing the

isolation resistor with a diode, whose current-voltage relation is the standard one for a Schottky diode device[8].

The voltage output of the circuit was solved numerically for a temperature range of -100 °C to 450 °C in MATLAB. The results are shown in Figure S7(c). Qualitatively the simulation results exactly agree with the measurement results, with the voltage output becoming nonlinear and then decreasing at around ~200 °C. This decrease in voltage is due to the temperature becoming high enough that the Schottky barrier allows significant current through, thus no longer behaving as an isolation resistance and ruining the principle of operation of the device. In addition, note that according to the circuit model there are no nonlinearities down to -100 °C, indicating that the minimum temperature of the device will only be limited by changes to the Seebeck coefficient of silicon at lower temperatures which are small.

Thus, the breakdown of the Schottky barrier at high temperatures limits the maximum temperature of the metal-on-silicon thermocouples to ~150 °C. Efforts to increase the height of the Schottky barrier cause higher temperatures to be required before breakdown of the barrier occurs, further extending the range. However, it should again be noted that for the majority of applications that require the extremely high thermal sensitivity that these metal-on-silicon devices offer such as biological applications, the excellent linearity from 20 to 80 °C is more than enough.

## Figures

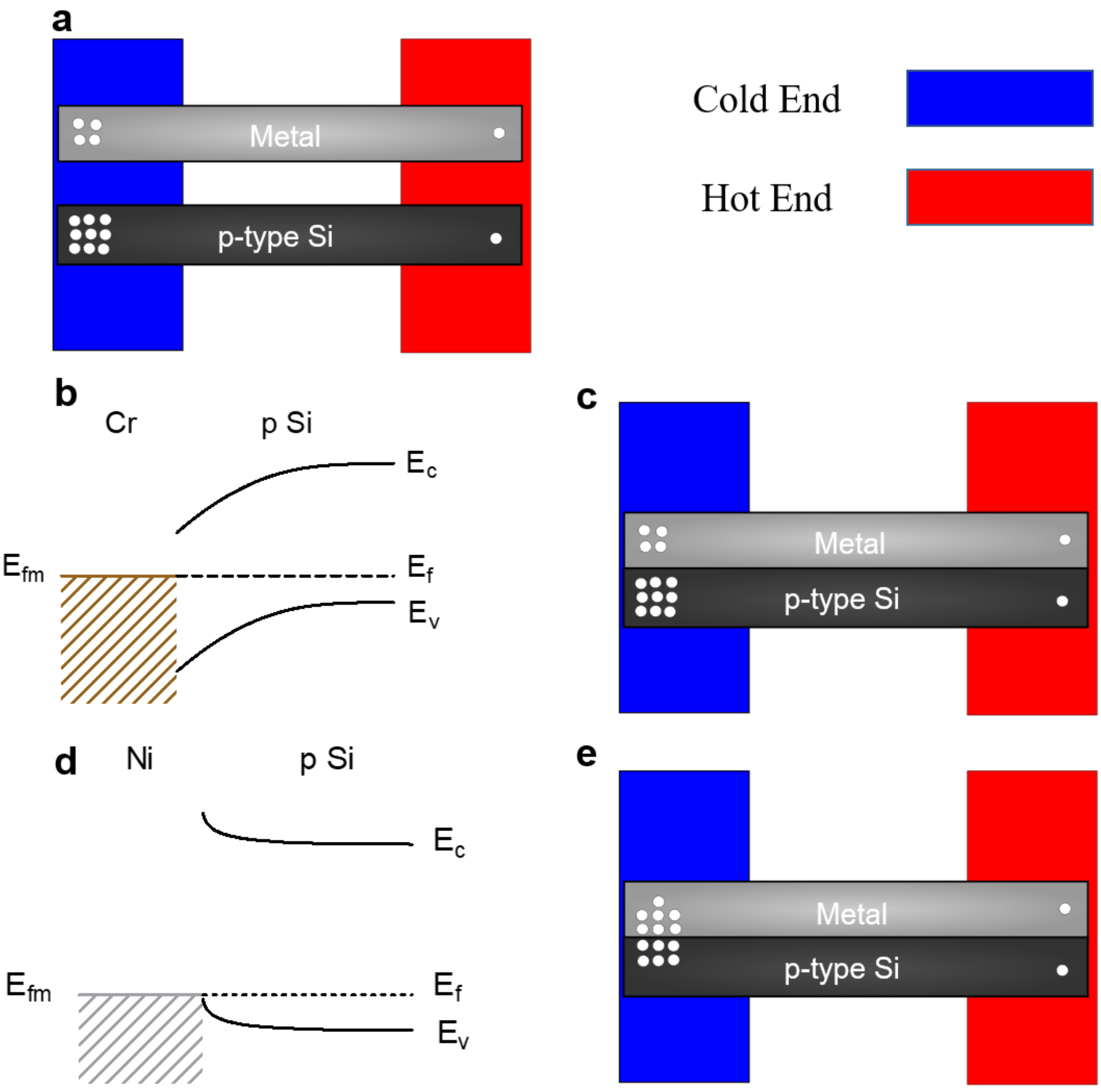


Figure S1: (a) Charge carrier diagram of the Seebeck effect when the hot side is heated and the cold side is cooled. Charge carriers (white circles) diffuse from the hot to the cold ends, creating a voltage across the material. Note that in the Si, there is a larger concentration of carriers diffusing,

resulting in a larger voltage. (b) Band diagram of an interface between p-doped silicon and a metal with a smaller work function than the silicon(Cr). Notice the band bending at the interfaces causes $E_v$ to be further away from $E_f$ at the interface, indicating the formation of a depletion region, called a Schottky barrier, which prevents current flow. (c) Charge carrier diagram when the metal is in contact with the p-Si and forms a Schottky barrier. Since charge carriers cannot conduct across the barrier, each material maintains its own charge carrier gradient and Seebeck generated voltage. (d) Band diagram of an interface between p-doped silicon and a metal with a larger work function than the silicon (Ni). Notice the band bending now causes $E_v$ to be slightly closer to $E_f$ at the interface, indicating no depletion region forming and thus current can flow easily between the two materials, forming an ohmic contact. (e) Charge carrier diagram when a metal is in contact with the p-Si, forming an ohmic contact. Since charge carriers can conduct across the barrier, the larger concentration of charge carriers in Si diffuses into the metal, leading to a larger concentration of charge in the metal and thus a larger generated voltage.

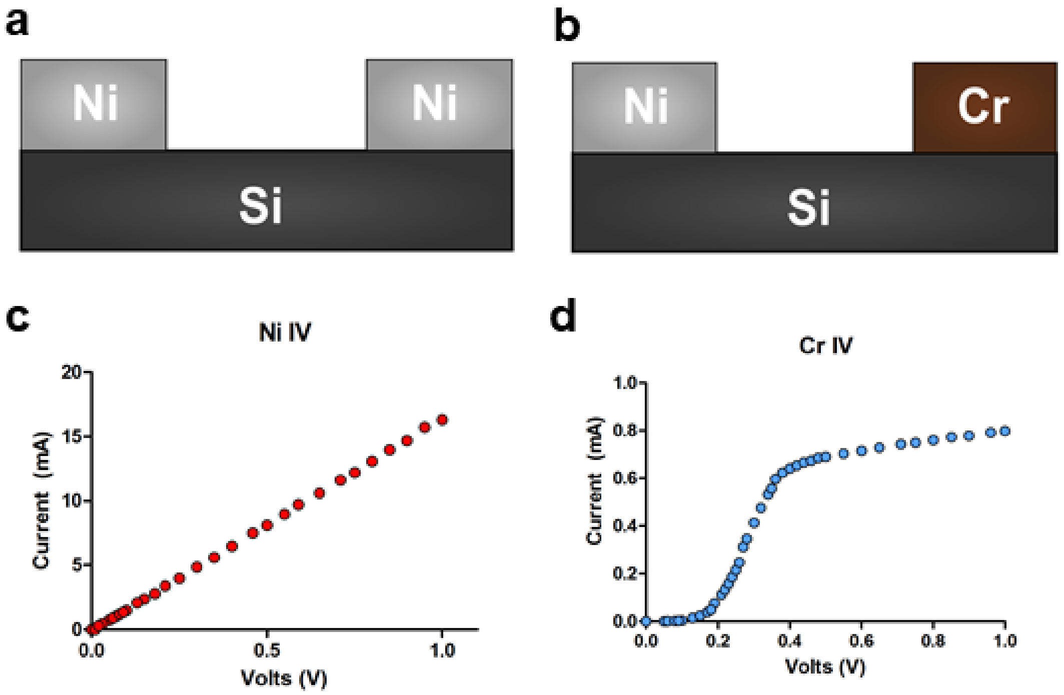


Figure S2: Current-voltage (IV) tests for thin metal strips on silicon (a) Schematic of the Ni pieces on the Si for the Ni IV measurement. A wire is placed on each Ni pad and the IV curve is measured between them. (b) Schematic of the Cr IV measurement setup. A wire is placed on the Ni and the Cr and the IV curve is measured between them. (c) The IV curve measured of two separate Ni pieces as shown in (a), demonstrating linearity which indicates an ohmic contact is formed. (d) The IV curve measured from the setup in (b). The rectifying curve indicating formation of a Schottky barrier.

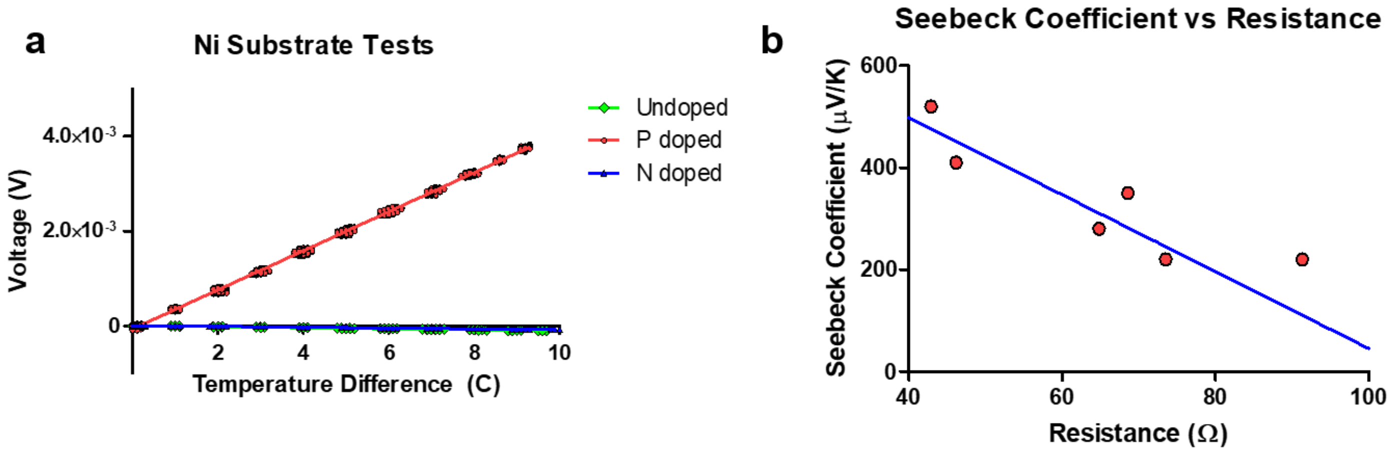


Figure S3: (a) Seebeck measurements of Ni on different substrates. Ni forms an ohmic contact only on the p-doped substrate, and thus it is the only one with the large Seebeck effect. (b)Seebeck coefficient of identical Ni pieces on differently-sized Si substrates versus the resistance of the Ni pieces. The theoretical prediction (blue line) is in good agreement with the data.

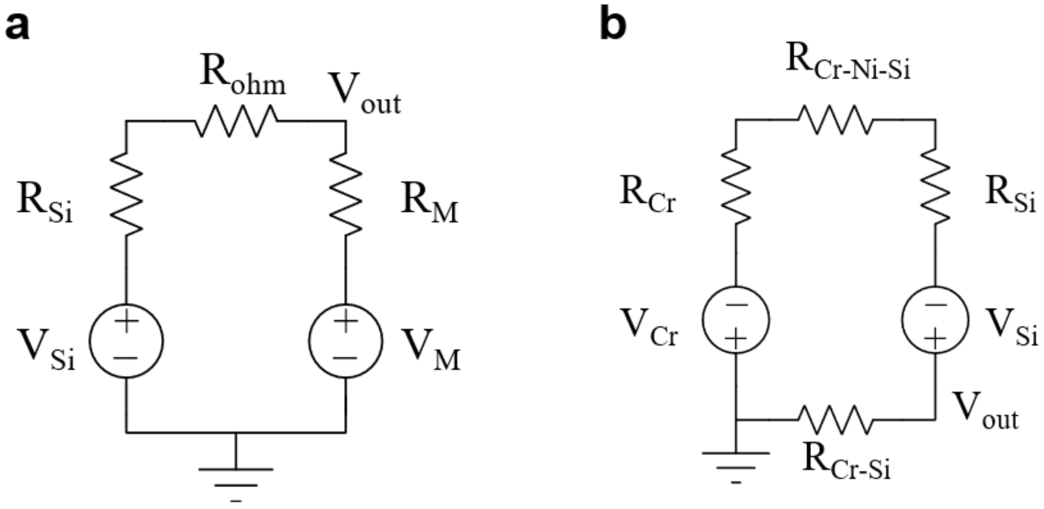


Figure S4: Equivalent circuits for circuit analysis. (a) Equivalent circuit for a single leg device. (b) Equivalent circuit for a single point device (please also refer to Figure 2).

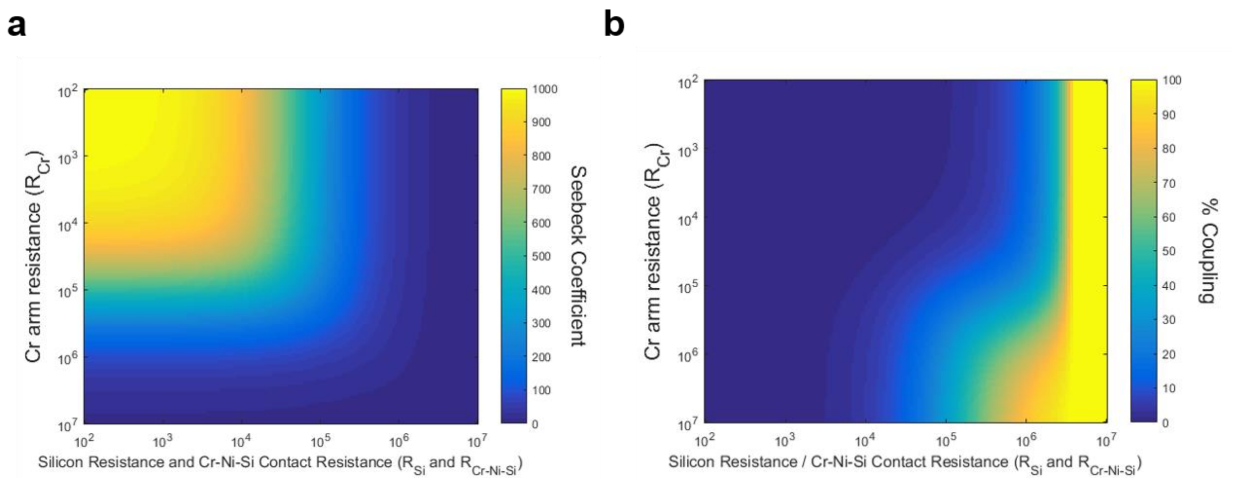


Figure S5: Results from the circuit simulation of two points on a shared substrate. (a) The overall Seebeck coefficient as a function of resistance of the Cr arm and Silicon and Cr-Ni-Si resistance. The coefficient quickly drops as either silicon's resistance increases or as the metal's resistance increases. (b) The amount of coupling between the two points. The coupling only becomes significant if the silicon's resistance becomes large compared to the Cr-Si Schottky barrier resistance. The graphs indicate that as long as the substrate's resistance and the Cr-Ni-Si resistance is kept low and the resistance of the Cr arm does not become high, the device should have close to ideal properties with no significant amount of coupling. The simulations assumed RSi = 1000 µV/K and $R_{Cr\text{-}Ni\text{-}Cr} = 10^5\ \Omega$.

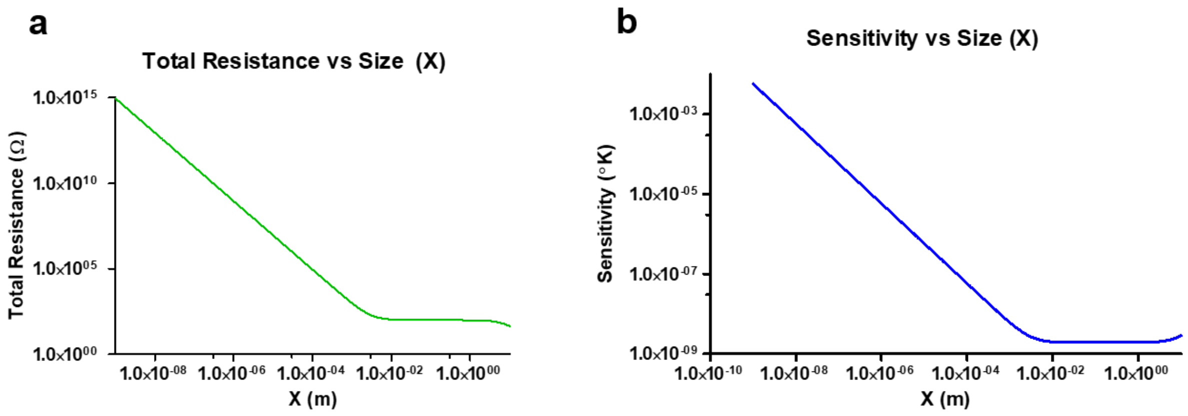


Figure S6: Analysis of potential for miniaturization. (a) The total resistance (measured between the nickel pad and the chrome pad on the cold end of the device) versus the characteristic size (x). The resistance of the device increases as the device becomes smaller, limiting the minimum size of the device at around 5 microns. If the resistance of the device becomes too high, the thermal voltage cannot be measured accurately. (b) Temperature sensitivity of the device (considering Johnson noise) versus the size: the sensitivity deteriorates as the device gets smaller due to an increase in resistance. Only Johnson noise was considered as this is the main source of noise that will increase as the resistance rises.

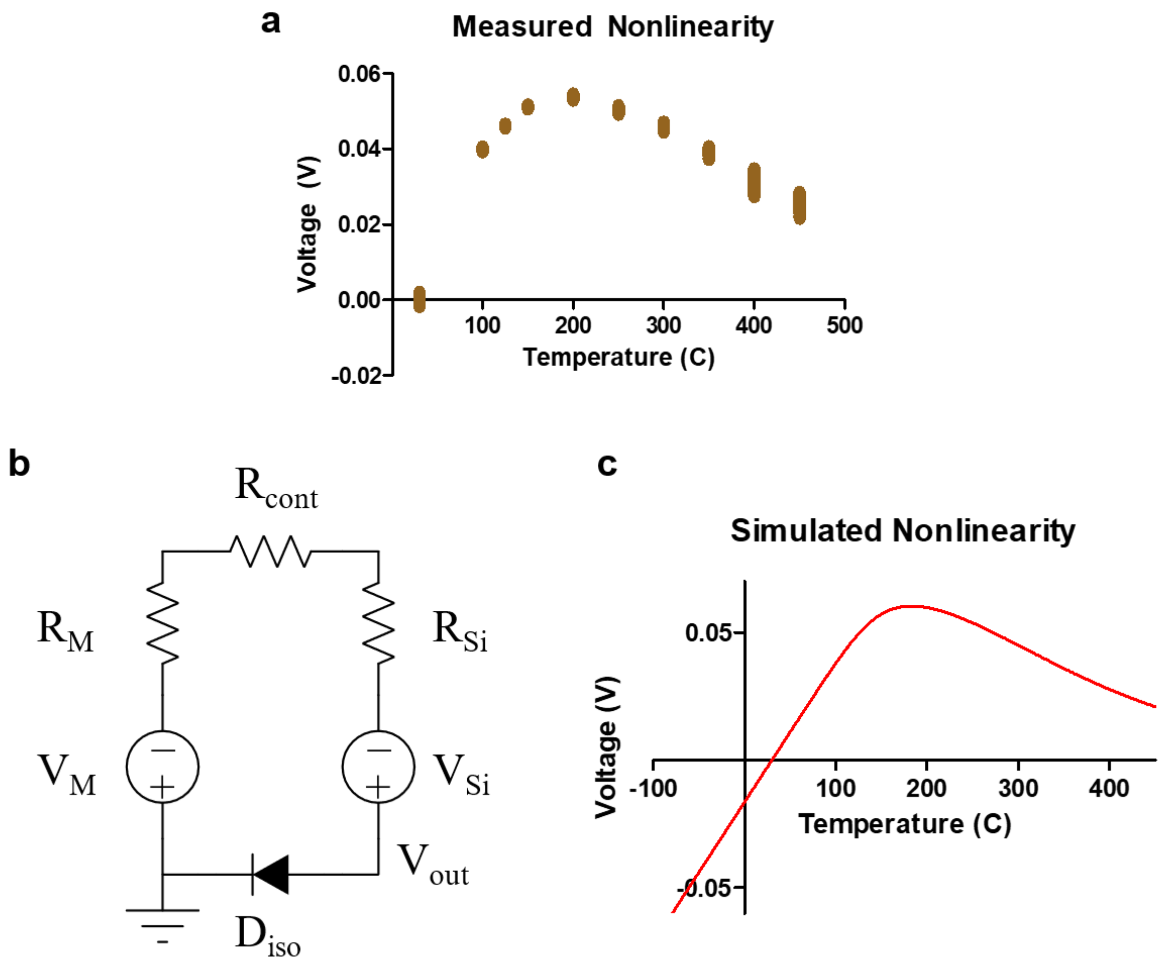


Figure S7: Nonlinearities over a larger temperature range. (a) The voltage output of the device measured beyond 100 °C. The voltage output begins to show nonlinearities as the temperature approaches 200 °C, and eventually begins to decrease. (b) The equivalent circuit model for the device where the isolation resistance is no longer modeled as a resistor but instead as a diode which takes into account the increasing conductance of the Schottky barrier at higher temperatures. (c) The simulated voltage response of the device qualitatively shows the same nonlinearity approaching 200 °C as well as no nonlinearity at subzero temperatures. This shows that the nonlinear output of the metal-on-silicon thermocouples is due to the breakdown of the electrical isolation of the Schottky barrier at higher temperatures.

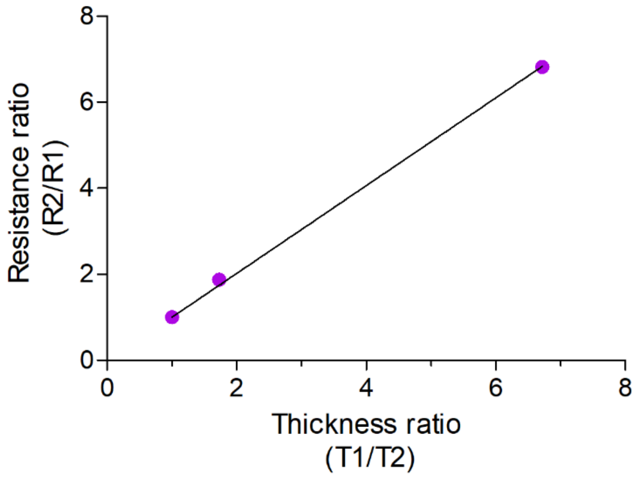


Figure S8: Change in wafer resistance with reduction in wafer thickness utilized for fabrication of flexible wafers. The x-axis represents the fractional decrease in wafer thickness (original thickness/changed thickness) whereas the y-axis represents fractional increase in the resistance (changed resistance/original resistance). The decrease in wafer thickness has a very close to linear correlation with increase in resistance ($R^2 = 0.995$) and the slight variation could be accounted to non-uniform wafer etching and edge effects.

## References


1. Richard S. Muller, T.I.K. *Device Electronics for Integrated Circuits*, Edn. 3rd. Wiley, New York, 2002).
2. Michaelson, H.B. The work function of the elements and its periodicity. *J. Appl. Phys.* **48**, 4729-4733 (1977).
3. Chang, C., Fang, Y. & Sze, S. Specific contact resistance of metal-semiconductor barriers. *Solid-State Electron.* **14**, 541-550 (1971).
4. Sze, S.M. & Ng, K.K. Metal-semiconductor contacts. *Phys. Semicond. Devices*, 134-196 (2006).
5. Blatt, F.J., Schroeder, P.A., Foiles, C.L. & Greig, D. *Thermoelectric power of metals*. (Plenum Press, New York, 1976).

6. Fulkerson, W., Moore, J.P., Williams, R.K., Graves, R.S. & McElroy, D.L. Thermal conductivity, electrical resistivity, and seebeck coefficient of silicon from 100 to 1300K. *Phys. Rev.* **167**, 765-782 (1968).

7. Jyothi, I. *et al.* Temperature dependency of Schottky barrier parameters of Ti Schottky contacts to Si-on-insulator. *Mater. Trans.* **54**, 1655 - 1660 (2013).

8. Colinge, J.-P. & Colinge, C.A. *Physics of semiconductor devices*. (Springer, New York, 2005).
